# A novel proteomic signature of osteoclast differentiation unveils the deubiquitinase UCHL1 as a necessary osteoclastogenic driver

**DOI:** 10.1038/s41598-024-57898-y

**Published:** 2024-03-27

**Authors:** Maria Materozzi, Massimo Resnati, Cecilia Facchi, Matteo Trudu, Ugo Orfanelli, Tommaso Perini, Luigi Gennari, Enrico Milan, Simone Cenci

**Affiliations:** 1 Age Related Diseases Unit, IRCCS Ospedale San Raffaele, Milan, Italy; 2 Università Vita-Salute San Raffaele, Milan, Italy; 3 Department of Medicine, Surgery and Neurosciences, University of Siena, Siena, Italy

**Keywords:** Cell biology, Proteome, Proteomics

## Abstract

Bone destruction, a major source of morbidity, is mediated by heightened differentiation and activity of osteoclasts (OC), highly specialized multinucleated myeloid cells endowed with unique bone-resorptive capacity. The molecular mechanisms regulating OC differentiation in the bone marrow are still partly elusive. Here, we aimed to identify new regulatory circuits and actionable targets by comprehensive proteomic characterization of OCgenesis from mouse bone marrow monocytes, adopting two parallel unbiased comparative proteomic approaches. This work disclosed an unanticipated protein signature of OCgenesis, with most gene products currently unannotated in bone-related functions, revealing broad structural and functional cellular reorganization and divergence from macrophagic immune activity. Moreover, we identified the deubiquitinase UCHL1 as the most upregulated cytosolic protein in differentiating OCs. Functional studies proved it essential, as UCHL1 genetic and pharmacologic inhibition potently suppressed OCgenesis. Furthermore, proteomics and mechanistic dissection showed that UCHL1 supports OC differentiation by restricting the anti-OCgenic activity of NRF2, the transcriptional activator of the canonical antioxidant response, through redox-independent stabilization of the NRF2 inhibitor, KEAP1. Besides offering a valuable experimental framework to dissect OC differentiation, our study discloses the essential role of UCHL1, exerted through KEAP1-dependent containment of NRF2 anti-OCgenic activity, yielding a novel potential actionable pathway against bone loss.

## Introduction

Bone health is a fundamental component of human well-being, whose relevance has been increasingly explored and acknowledged over the last 20 years. Deranged bone homeostasis is associated with aging and many different disorders. Indeed, skeletal and bone metabolic alterations occur in diverse high-impact pathologies, including age-related diseases, metabolic or hormonal imbalance, genetic disorders, and cancer^1–4^. The predominant consequence of such alterations is bone loss, a feature of highly prevalent diseases like osteoporosis, diabetes, or metastatic cancer, with remarkable impact on public and individual health^1,4–8^. Indeed, the resulting skeletal damage inevitably leads to bone pain and fragility fractures that, in Europe alone, have been estimated to occur at the rate of around 2.7 million per year. Moreover, the yearly incidence of fragility fractures is expected to rise to ~ 3.3 million by 2030 due to demographic aging, with decreased quality of life and increased morbidity, disability, and healthcare costs^8^.

Bone loss is invariably mediated by heightened differentiation and/or activity of osteoclasts (OC), specialized bone-resorbing cells that originate from hematopoietic precursors through a complex differentiation process, referred to as osteoclastogenesis (OCgenesis), in which they fuse to generate giant multinucleated syncytia capable of degrading the bone matrix. OCgenesis is driven and regulated by diverse cues, among which the key pro-OCgenic cytokine RANKL, produced mainly by bone and immune cells. In physiological conditions, bone homeostasis is maintained by a variety of cellular populations, hormones, and mechanic stimuli that concertedly ensure efficient controlled remodelling, balancing bone formation by osteoblasts and bone resorption by OCs^9,10^.

In disease, however, the disruption of this balance, due to metabolic, hormonal, or genetic alterations generally results in deregulated bone resorption by OCs, leading to net bone loss and skeletal fragility^1,11–13^. Osteolysis also plays a major role in cancer, being bone a paradigmatic metastatic site in which neoplastic cells recruit and promote OCs to resorb bone and sustain tumour growth—a deleterious vicious cycle therapeutically contrasted by blocking the generation and resorptive activity of OCs, e.g., using anti-RANKL mAb and bisphosphonates^4,12–17^.

Despite OCs being recognized as central to many diseases, and OC biology being increasingly investigated, the molecular mechanisms regulating their differentiation and resorptive activity are still partly elusive and warrant further investigation. Moreover, the recent recognition of heterogeneity in OC ontogeny and of new immunoregulatory functions urges to explore new roles and potential targeting strategies^18–21^. Studying OC biology is therefore essential to pursue a better understanding of physiological bone homeostasis and its pathologic derangement and to devise new therapies against bone loss across diseases.

Building on this background, we conducted a twofold unbiased proteomic study of primary murine in vitro OCgenesis, to explore regulatory mechanisms and uncover new potential therapeutic targets for skeletal diseases. To do this, we comprehensively evaluated the proteome reshaping of RANKL-dependent OC differentiation from mouse purified bone marrow monocytes (BMM), adopting two independent quantitative comparative proteomic approaches. Our study identified profound proteome changes, entailing expansion of OC-specific pathways and contraction of macrophage/immune counterparts, and reorganization of cellular compartments collectively aimed at resorbing bone. Since most gene products are currently unannotated in OC-related functions, our data define a novel protein signature of OCgenesis. Moreover, our work highlights the deubiquitinase UCHL1 as the most upregulated cytosolic protein in OCs and proves it essential for OCgenesis through a novel mechanism modulating the anti-OCgenic activity of the transcriptional activator NRF2 in a redox-independent manner.

## Results

### Unbiased characterization of OC differentiation by proteomics

**Figure 1:**
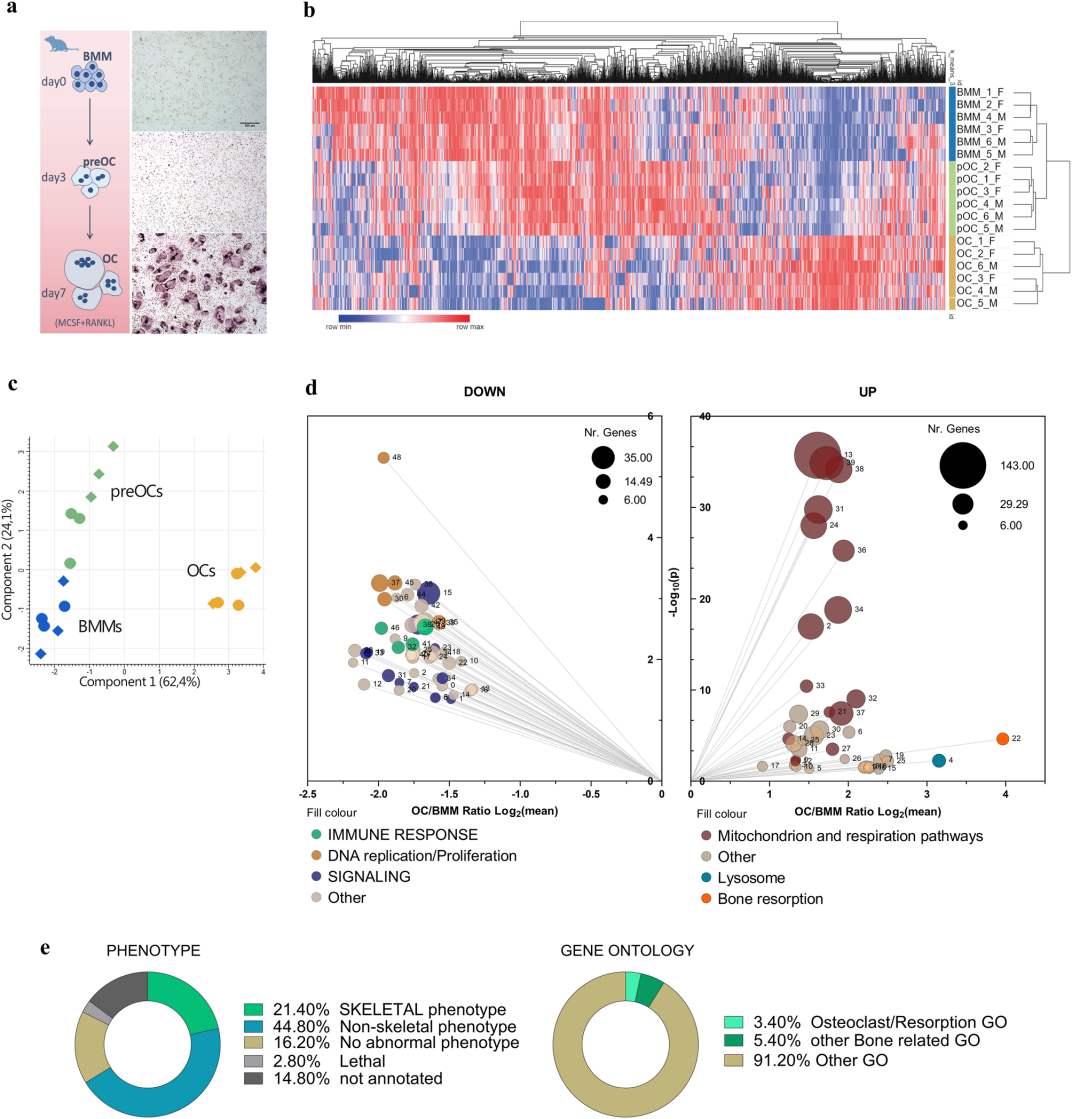
Label-free quantitative proteomics of OC differentiation. (**a**) Experimental framework. Left: schematic representation of the timepoints at which differentiating OCs were studied by proteomics; right: corresponding representative TRAP-stained images of bone marrow monocytes (BMM), preOCs and OCs (scale bar: 500 µM); (**b**) Hierarchical and k_means clustering of label-free MS/MS proteomics in BMMs, preOCs (pOC), and OCs; **c)** Principal component analysis of label-free quantitative proteomics at the indicated steps during OCgenesis; circles and diamonds indicate respectively female and male samples; (**d**) Gene ontology (GO) enrichment analysis of the OC proteome compared to BMM by ClueGO showing most depleted (left) and enriched (right) pathways; (**e**) Association of the identified OC DEPs with skeletal phenotypes according to the Mouse Genome Informatics (MGI) database (left) and Gene Ontology (GO) annotations (right).

**Figure 2:**
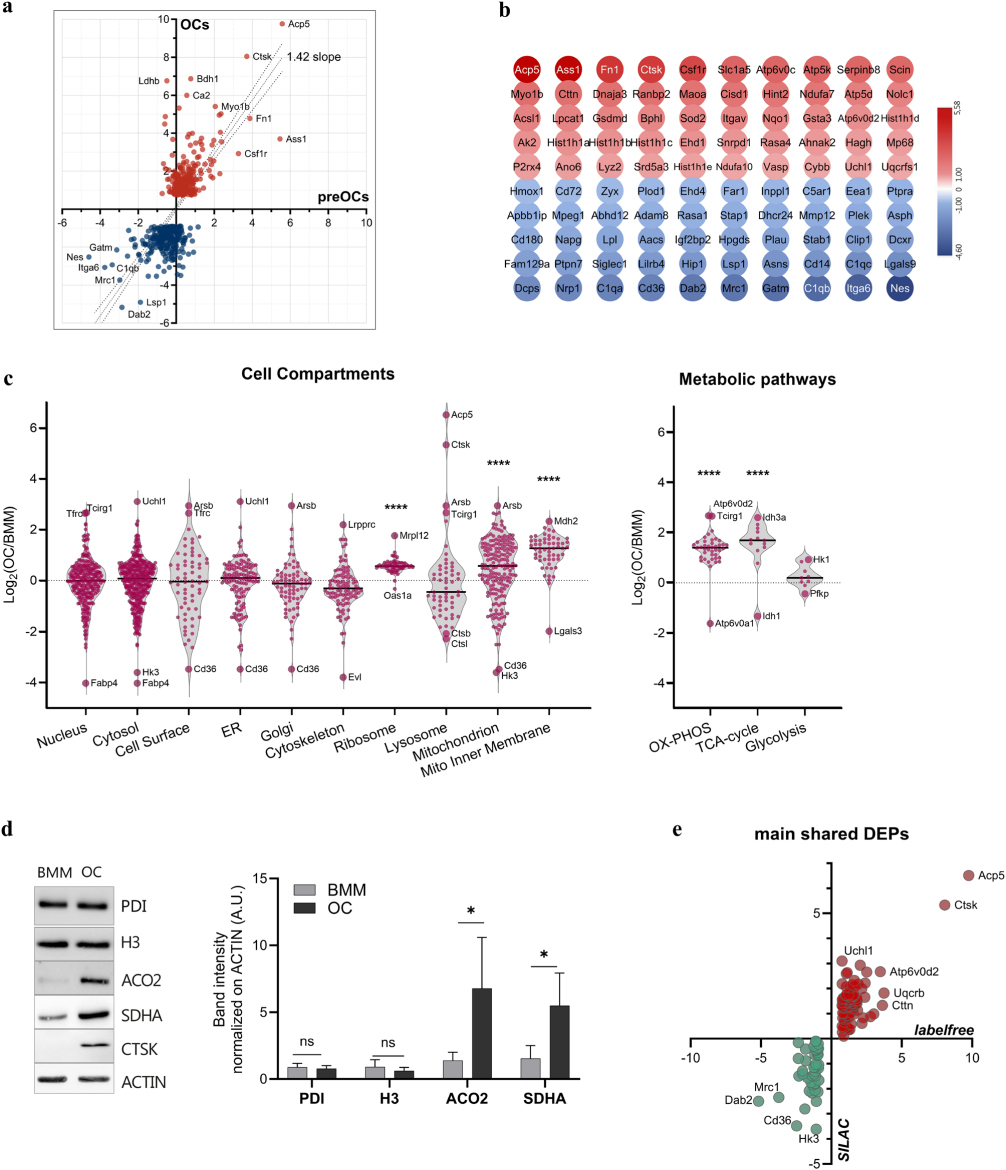
Validation of OC-specific proteomic signature by SILAC proteomics. (**a**) Protein expression of the identified top 500 DEPs in preOCs and OCs (average ratio vs BMMs, n = 6, linear regression test) showing early and consistent modulation; (**b**) OCgenic protein signature (gene names), defined as the top 100 consistent DEPs in preOCs and OCs. Colour coding indicates protein expression ratio in pOCs/BMMs (log_2_); (**c**) Proteins quantified by SILAC proteomic analysis grouped by the indicated GO categories: cell compartments (left) and metabolic pathways (right). Panels show average log_2_ OC/BMM protein expression ratios for each category (one-way ANOVA with Dunnett's multiple comparisons test, ****p < 0.0001); (**d**) Immunoblot analysis of selected proteins in BMMs and OCs. Left: Representative blot images of the indicated proteins, representative of the following organelles: ER (PDI), nucleus (H3), mitochondria (SDHA and ACO2). Right, quantification of band intensities of at least 3 independent experiments normalized on actin (mean ± SD; paired *t* test, ∗ *p* < 0.05); (**e**) Overlap of SILAC/label-free DEPs: 130 proteins consistently differentially regulated in both SILAC and label-free top 500 DEPs (log2).

To comprehensively define the cellular and molecular changes that occur during primary murine OCgenesis, we designed and performed integrated label-free proteomic studies at different timepoints along RANKL-dependent OC differentiation to compare the proteome of BMMs, preOCs and mature OCs (Fig. 1A). Hierarchical and k_means clustering analyses of the identified and quantified proteins successfully clustered three expected datasets corresponding to BMMs, i.e., OC precursors not exposed to RANKL, preOCs, i.e., BMMs treated with RANKL for 3 days, and OCs, i.e., cultures treated with RANKL for 7 days and rich in large multinucleated cells. The 3 clusters had distinct protein expression profiles, as shown by heatmap (Fig. 1B and Suppl. Dataset 1) and principal component analysis (PCA) (Fig. 1C). After filtering and optimization of the identified peptides, we found 1,476 statistically significant differentially expressed proteins (DEPs) in OCgenesis and investigated their function through gene ontology (GO) annotation and pathway enrichment analysis. The 500 most deregulated (top 250 upregulated and top 250 downregulated) proteins in OCs compared to BMMs were selected for pathway enrichment analyses. Upregulated proteins were significantly enriched for 240 pathways clustered in 40 groups (summarized in Fig. 1D, right panel, and Suppl.Fig. 1). Consistent with OC ontogeny, the highest mean expression increases were identified in the process of bone resorption and localization to lysosome, mainly driven by heightened concentration of specific proteases, phosphatases, and lysosomal ATPases known to be necessary for OC activity, such as CTSK, ARSB, TCIRG. However, the two largest pathway clusters were mainly accounted for by mitochondrial proteins (143 species, contributing 26% of identified pathways) mostly belonging to mitochondrion localization and oxidative phosphorylation (OXPHOS), highlighting a concerted, remarkable (~ threefold) increase in the intracellular abundance of the mitochondrial proteome during OC differentiation. Enriched pathways also included several processes related to cell motility, migration, adhesion, and cytoskeletal reorganization (Suppl.Fig. 1 and Dataset 2, and detailed description therein). Downregulated proteins were significantly enriched for 170 pathways clustered in 50 groups (Fig. 1D, left panel, and Suppl.Fig. 1). Many downregulated pathways overlapped with functions related to immune response (e.g., regulation of innate immune response, regulation of leukocyte), cell replication, and chromosome organization, together with several cellular signalling pathways (e.g., response to hydrogen peroxide, ERBB signalling, TNF production; see detailed description in Suppl.Fig. 1 and Dataset 2). Collectively, the breadth and depth of these protein expression changes attest to a profound metamorphosis driving OC differentiation, coherent with the transition from the innate immune functions and high replicative potential typical of the monocytic-macrophage lineage to the high energy production and lysosomal secretion of lytic enzymes required for bone degradation. Of note, protein expression changes during OCgenesis in cells from female and male mice were largely superimposable (Fig. 1B,C), except for small differences in preOCs and OCs, mainly accounted for by proteins with slight expression changes (Suppl.Fig. 2B). Pathway analysis for sex-dependent gene products identified significantly different processes primarily in preOCs, pertaining mainly to metabolism, while OC expression profiles appeared consistent among genders, except for small differences in proteins involved in antigen processing and presentation (Suppl.Fig. 2C; Suppl. Dataset 2).

Notably, a sizeable portion of the top DEPs (21.4%) were protein products of genes associated with skeletal-related phenotypes (e.g., bone deformities, increased or decreased bone mass) in the Mouse Genome Informatics (MGI) database^22^, attesting to the relevance of the identified species to skeletal development and function (Fig. 1E, left panel). However, only 17 (3.4%) of the identified top 500 DEP-coding genes in OCs are annotated in GO pathways associated with OC identity or bone resorption, and a mere 5.4% (27 genes) in GO bone-related pathways; the remaining genes, encoding the vast majority of proteins most deregulated in OC differentiation, are not currently associated with OCs and skeletal traits (Fig. 1E, right panel), suggesting the existence of previously unrecognized biological functions of these proteins integrated with the originally annotated ones.

Most DEPs in OCs were found to be already sizeably and significantly differentially expressed early in OCgenesis, i.e., prior to the appearance of large syncytia resembling mature OCs (Fig. 2A, Suppl.Fig. 2A). Thus, our data describe a novel protein signature of OCgenesis, of which the 100 species found most differentially expressed from early to terminal OC differentiation are shown in Fig. 2B (Suppl.Dataset 1). To challenge and validate our findings, we deployed an additional proteomic approach wherein we compared mature OCs with BMMs by SILAC, a distinct established technique affording a more quantitatively accurate identification of DEPs by incorporation of stable isotope-labeled amino acids in newly synthesized proteins, enabling ratiometric protein quantification in a single mass spectrometry run. Validating our previous observations, SILAC proteomics confirmed a steep increase in the abundance of mitochondrial proteins, with distinctively enhanced expression of enzymes catalyzing OXPHOS and the tricarboxylic acid (TCA) cycle. This approach also confirmed a profound lysosomal reshaping, with the expected increased abundance of OC-specific proteases and phosphatases, despite overall lysosomal protein content was not higher, on a per protein basis, in OCs as compared to BMMs, as shown by cell compartment analysis (Fig. 2C). The increased concentration of selected proteins representative of major cell compartments was also confirmed by targeted immunoblotting experiments (Fig. 2D). Notably, overlap of the top DEPs identified by the two independent proteomic approaches, i.e., label-free and SILAC, showed consistent results and comparable fold expression changes for most proteins, including OC-specific species (e.g., CTSK, ACP5, TCIRG, ATP6v0d2) (Fig. 2E).

Overall, our studies concordantly identify a novel protein signature, exemplified by the top 100 species differentially expressed from early to terminal OC differentiation. The major changes occurring in OCgenesis capture remarkable increases in mitochondrial mass and metabolic function and relevant qualitative changes in the lysosomal compartment, coupled to decreased immunological functions and replicative potential, with deregulation of specific signalling and immune-related pathways. Taken together, our data identify a protein signature of murine OCgenesis, detected early in differentiation, involving gene products largely unannotated in bone biology.

### Identification of UCHL1 as a novel essential driver of OCgenesis

The SILAC proteomic analysis of OCgenic reshaping highlighted, among other species, ubiquitin carboxyl-terminal hydrolase isozyme L1 (UCHL1) as the protein that showed the strongest (~ ninefold) increase among cytosolic proteins in OCs as compared to BMMs (Fig. 2C), concordantly captured in the OCgenic signature identified in our label-free analysis (Fig. 2B,E). UCHL1 is a deubiquitinating enzyme (DUB) known to be highly expressed in neurons and previously implicated in neurodegeneration and several forms of cancer, but poorly characterized in bone biology^23–28^.

**Figure 3:**
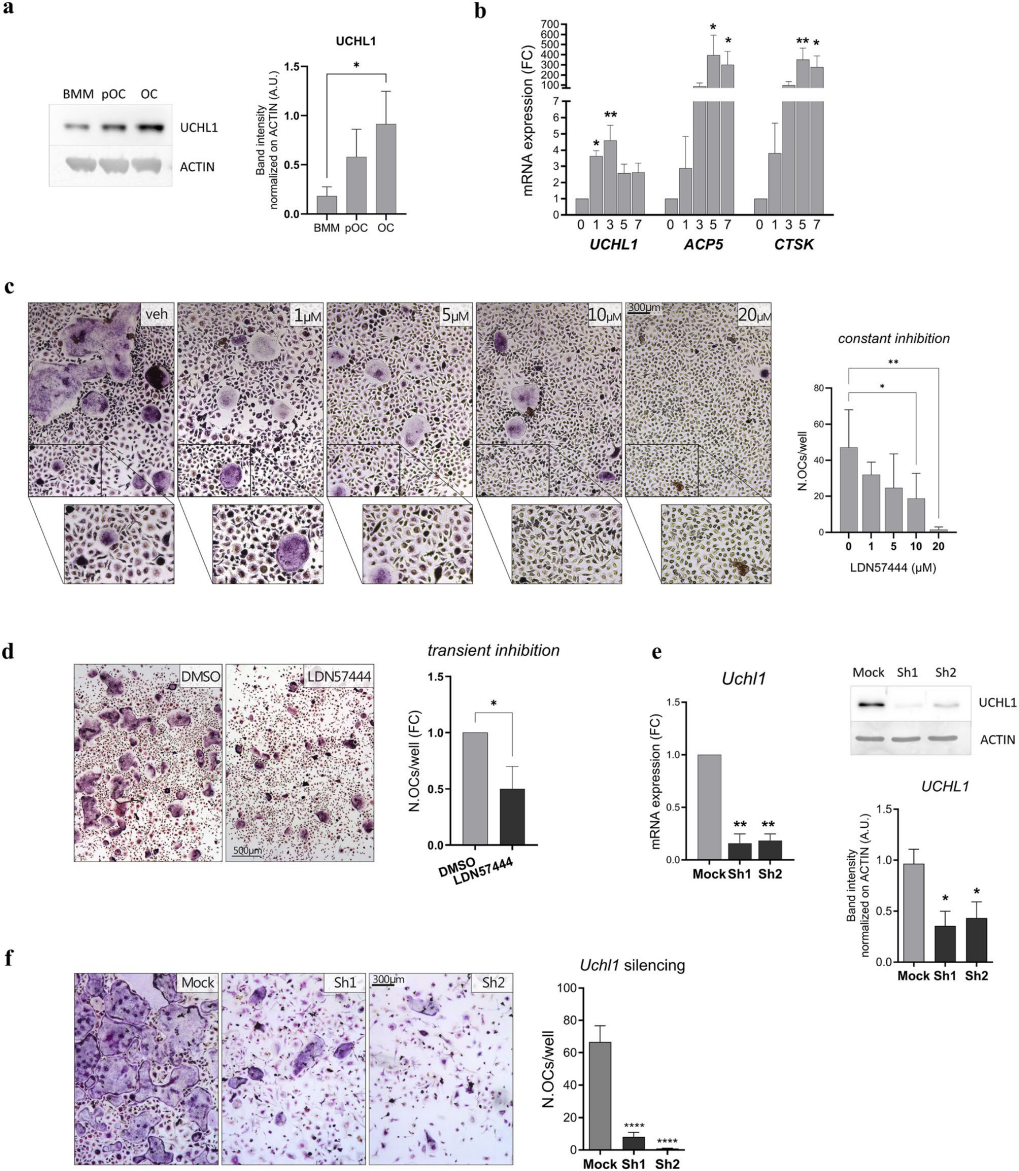
Essential role of UCHL1 in OC formation. (**a**) Immunoblot analysis of UCHL1 protein abundance during OCgenesis. Left, representative images; right, quantification of band intensity normalized on actin (mean ± SD, n = 3 independent experiments, Kruskal Wallis test with Dunn multiple comparison vs BMMs, *p < 0.05); (**b**) qRT-PCR analysis of *Uchl1, Acp5, and Ctsk* mRNAs during OCgenesis (mean ± SD normalized on actin mRNA; n = 3 independent experiments, Kruskal Wallis test with Dunn multiple comparison vs BMMs, *p < 0.05, **p < 0.01); (**c**) Effect of continuous pharmacological inhibition of UCHL1 by LDN57444 at the indicated doses (administered together with RANKL at the start of differentiation and at every change of medium) or vehicle (DMSO) on OC formation as assessed by TRAP staining. Left, representative images; right, quantification of the number of OCs per well (mean ± SD, n = 3 independent experiments, RM one-ANOVA test with Dunnett’s multiple comparison vs BMMs, *p < 0.05, **p < 0.01); (**d**) Effect of transient pharmacological inhibition of UCHL1 by LDN57444 at day 3 of differentiation (24 h, 20 µM) on OCgenesis as assessed by TRAP staining. Left, representative images (scale bar 500 µm); right, quantification of the number of OCs per well (mean ± SD, n = 4 independent experiments, Welch’s *t*-test, *p < 0.05); (**e**) Analyses of *Uchl1* expression in silenced preOCs (sh1, sh2) vs control (mock) by qRT-PCR (left, mean ± SD, n = 3 independent experiments, Welch’s *t*-test) and immunoblot (right, mean ± SD, n = 4 independent experiments, paired *t*-test, *p < 0.05); (**f**) Effect of Uchl1 silencing by lentiviral expression of specific (sh1, sh2) or mock shRNAs on OC formation assessed as above. Left, representative images; right, quantification of the number of OCs per well (mean ± SD, n = 3 independent experiments, paired *t*-test, ****p < 0.0001).

Consistent with a role in OCgenesis, Coudert et al. reported higher expression of *Uchl1* mRNA in patients with autosomal dominant osteopetrosis type II, a rare inherited disorder characterized by high bone mass, in which OCs are increased in number, but incapable of resorption^29^. Immunoblot experiments confirmed that UCHL1 protein abundance increases significantly and progressively during OC differentiation (Fig. 3A). Moreover, qRT-PCR analyses revealed that *Uchl1* transcripts are strongly induced upon OCgenesis, with a significant rise very early in differentiation, i.e., already at day 1 following RANKL stimulation, and maintained thereafter until OC maturation (Fig. 3B). Notably, within the monocyte lineage, UCHL1 induction appeared specific to OC differentiation, since its protein abundance did not increase, but in fact decreased or remained stable in BMMs respectively polarizing towards M1/M2 macrophage phenotypes (Suppl.Fig. 3A). Based on its distinctive and specific expression pattern, we postulated a relevant role of UCHL1 in OC differentiation and activity and challenged our hypothesis by blocking UCHL1 during in vitro OC differentiation both pharmacologically, through a specific small molecule inhibitor, LDN57444^30^, and genetically, by lentiviral stable shRNA expression. Treatment of RANKL-stimulated BMMs with nontoxic doses of LDN57444, following confirmation of lack of toxicity in mouse primary BMMs (Suppl.Fig. 3B–D), induced a significant and dose-dependent reduction of OCs as compared to vehicle-treated samples (Fig. 3C). Moreover, transient inhibition, achieved through administration of the inhibitor for 24 h during the third day of differentiation, resulted in significantly reduced numbers of mature OCs thereafter (Fig. 3D). Consistently, genetic inhibition of *Uchl1* through stable expression in BMMs of two different anti-*Uchl1* shRNAs, also induced a remarkable reduction in OC formation (Fig. 3E,F). Overall, the data reveal an essential role played by UCHL1 in OC-genesis, consistent with its distinctive expression, exerted already in early phases of differentiation.

### UCHL1 does not regulate ubiquitin-mediated protein homeostasis in differentiating OCs

**Figure 4:**
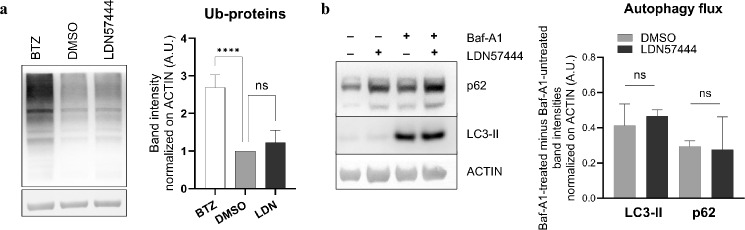
Effect of UCHL1 inhibition on ubiquitinated proteins and autophagy in differentiating OCs. (**a**) Immunoblot analysis of ubiquitin-conjugated proteins in preOCs upon treatment with LDN57444 (LDN, 20 µM, 72 h), bortezomib (Btz, 1 µM, 6 h), or vehicle (DMSO). Left, representative images; right, quantification of band intensities normalized on actin (mean ± SD, n = 3 independent experiments, one-way ANOVA with Dunnet’s multiple comparison test, ****p < 0.0001); (**b**) Assessment of autophagic flux estimated as the rate of lysosomal digestion of lipidated LC3 (LC3-II) or p62. Left, representative immunoblot analysis of LC3-II and p62 in preOCs treated with LDN57444 (20 µM, 72 h) or vehicle (DMSO) and the distal autophagy inhibitor bafilomycin-A1 (Baf-A1, 20 nM, 24 h). Right, autophagic flux of LC3-II and p62 estimated as actin-normalized band intensity difference between Baf-A1-treated and untreated samples (mean ± SD, n = 3 independent experiments, paired *t*-test).

In neurobiology, UCHL1 is known to play a key role as a multi-target DUB (pan-DUB) affording efficient ubiquitin (Ub) recycling for post-translational modification and subsequent degradation of Ub-conjugated proteins, hence ensuring cellular protein homeostasis (proteostasis)^31–37^. Since OCs have been shown to mount an adaptive unfolded protein response (UPR), typically activated to cope with the accumulation of misfolded and unfolded proteins in the endoplasmic reticulum (ER)^38^, we hypothesized that during OCgenesis UCHL1 elevation may serve to afford proteostasis sustaining the cellular availability of free Ub, required for efficient protein catabolism via the Ub proteasome system (UPS) and macroautophagy (conventionally, autophagy). However, UCHL1 inhibition did not result in significant accumulation of Ub-conjugated proteins in OCs, in contrast with the hypothesized pan-DUB role in these cells (Fig. 4A). Since autophagy cooperates with the UPS for the clearance of Ub-proteins, we then asked if UCHL1 inhibition results in a sizeable increase in autophagic workload, thereby affecting autophagic activity. However, UCHL1 inhibition did not impair the overall autophagic flux, as demonstrated by superimposable rates of lysosomal digestion of the autophagosome-decorating phosphatidylethanolamine-conjugated protein, LC3-II and the prototypical autophagic adapter and substrate, SQSTM1/p62 upon treatment with LDN57444 (Fig. 4B). Overall, these experiments indicate that UCHL1 does not act as a pan-DUB and does not afford cellular proteostasis in OC differentiation.

### UCHL1 promotes OCgenesis by restricting NRF2-dependent gene expression

**Figure 5:**
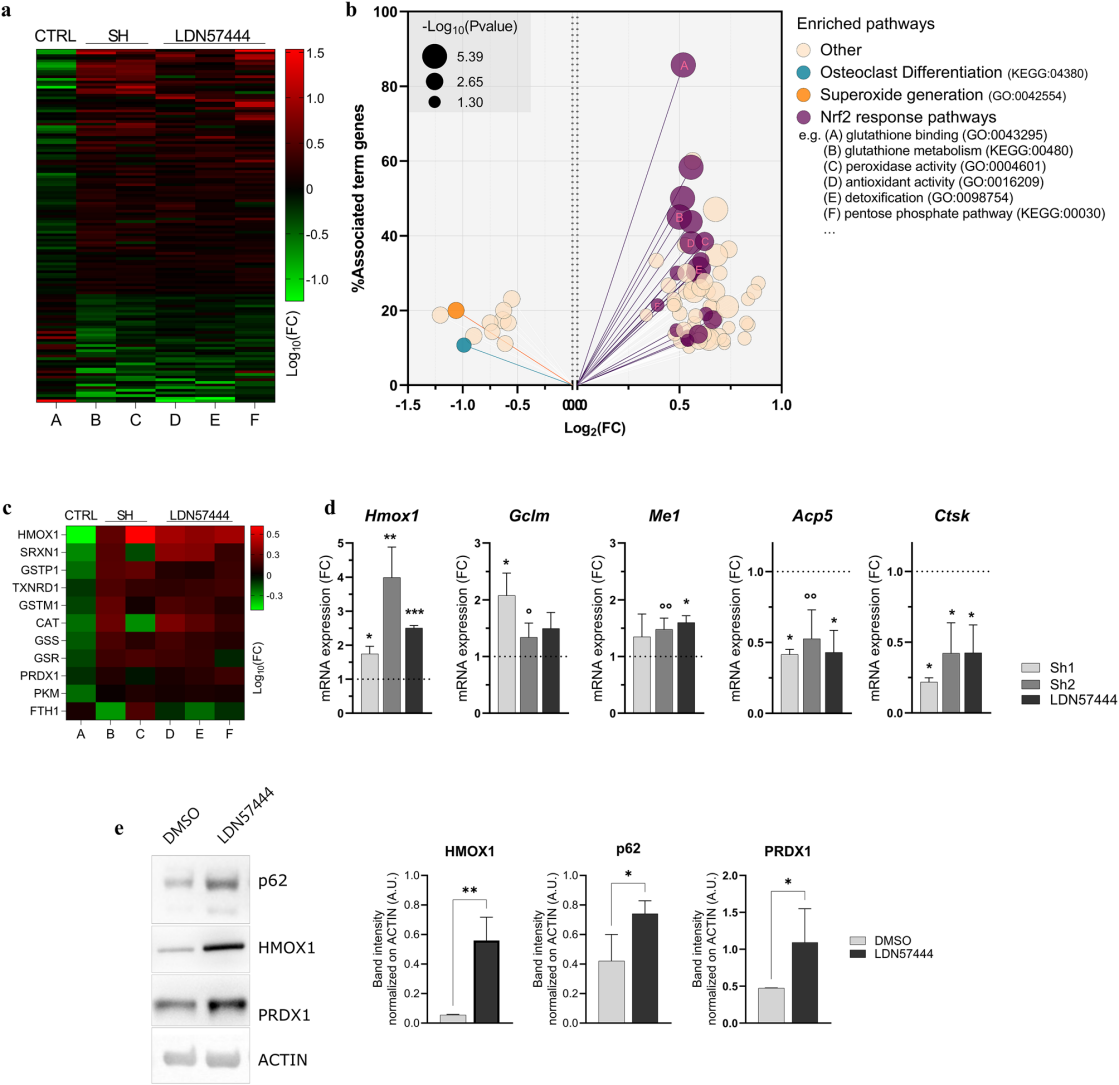
Heightened NRF2 activity is associated with suppression of OCgenesis by UCHL1 inhibition. (**a**) Heatmap of label-free proteomic analysis of BMMs and preOCs following genetic or pharmacologic UCHL1 inhibition. Log_10_FC of shared deregulated proteins in control OCgenesis (preOCs vs BMMs, column A), preOCs of shUCHL1 vs mock (columns B-C, Sh1, Sh2 respectively), and LDN57444 (20 µM, 72 h) versus vehicle (DMSO) in 3 independent replicates (columns D–F); (**b**) Gene ontology (GO) enrichment analysis of the shared DEPs by ClueGO showing enriched (right) and depleted (left) pathways; (**c**) Heatmap of NRF2 targets; Log_10_FCs as described in (**a**); (**d**) qRT-PCR analysis of the mRNA levels of NRF2 targets and OC canonical genes upon silencing or pharmacological inhibition (LDN57444 20 µM, 72 h) of UCHLI in preOCs (mean ± SD normalized on actin mRNA; n = 3–4, Welch’s *t*-test, *p < 0.05, **p < 0.01, °*p* = 0.07, °°*p* = 0.05); (**e**) Immunoblot analysis of NRF2 targets in preOCs treated with LDN57444 or vehicle (DMSO) for 72 h. Left, representative images; right, quantifications of band intensities normalized on actin (mean ± SD, n ≥ 3 independent experiments, unpaired *t*-test, *p < 0.05, **p < 0.01).

Having ruled out a general proteostatic function of UCHL1 in OCgenesis, to explore its role in differentiating OCs and understand the mechanism whereby UCHL1 supports OC differentiation, we deployed an unbiased approach through comprehensive label-free proteomics upon UCHL1 genetic and pharmacological inhibition in preOCs, achieved respectively via stable expression of anti-*Uchl1* shRNAs and administration of LDN57444. Out of 1,830 gene products detected in all samples (Suppl.Dataset 3), we focused on 135 proteins that were concordantly differentially expressed upon both genetic and pharmacological anti-OCgenic treatments. As summarized in the heatmap in Fig. 5A, the expression pattern of shared DEPs upon genetic (columns B–C) or pharmacologic (columns D–F) inhibition was opposite to that of control OCgenesis (column A), in full consistency with the observed profound impairment of OC differentiation achieved with both treatments. While analysis by cell compartment showed no concordant deregulation of entire organelles or compartments (Suppl.Fig. 4A), gene enrichment analysis of DEPs shared by the two inhibition strategies, besides the expected downregulation of OC differentiation, concordantly highlighted significant upregulation of pathways that belong to the canonical NRF2-dependent antioxidant gene expression program^39,40^, such as glutathione binding, glutathione metabolism, peroxidase activity, antioxidant activity, detoxification, and pentose phosphate pathway (Fig. 5B). Further attesting to heightened NRF2 activity, the protein pattern also entailed significantly lower expression of proteins related to the superoxide generation pathway (Fig. 5B). The enrichment of NRF2 targets was driven by significantly increased abundance of protein products of major NRF2 target genes, e.g., *Hmox1*, *Gstp1, Cat, Gss, Gsr, Prdx1*, as shown by the heatmap in Fig. 5C (see Suppl.Fig. 4B and Dataset 4 for a complete list of deregulated genes and pathways). To ascertain the impact of UCHL1 inhibition on NRF2 activity, we next evaluated the expression of representative NRF2 transcriptional targets upon UCHL1 genetic or pharmacological inhibition at both transcript and protein levels. In agreement with the observed proteomic characterization, qRT-PCR and immunoblot analyses showed significant upregulation of NRF2 targets upon UCHL1 blockade, with HMOX1 mRNA and protein showing the highest increase (Fig. 5D-E).

**Figure 6:**
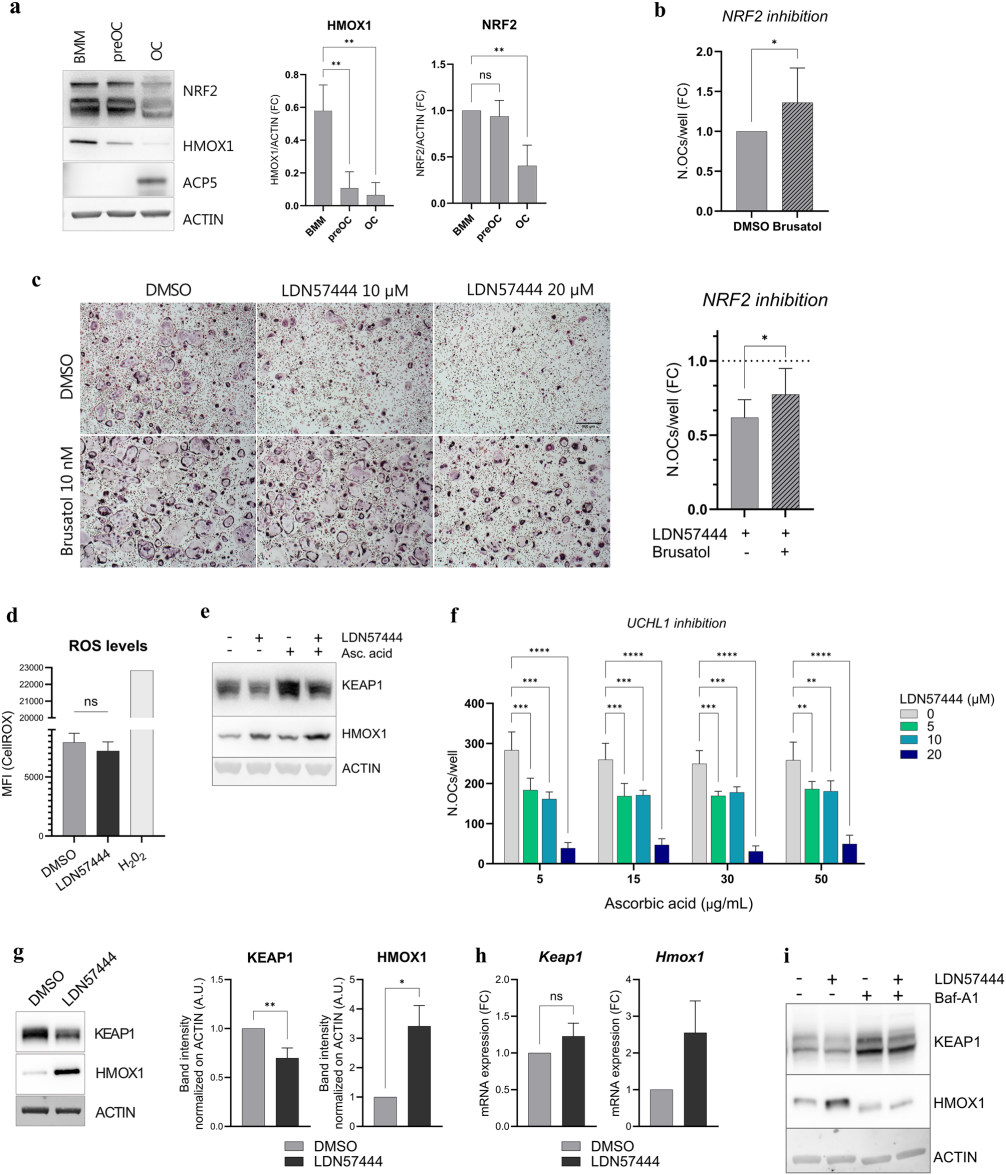
Redox-independent role of the KEAP1/NRF2 axis in UCHL1-dependent control of OCgenesis. (**a**) Immunoblot analysis of NRF2 targets during OCgenesis: representative images (left); quantifications of band intensities normalized on actin (right) (mean ± SD, n = 3 independent experiments, one-way ANOVA with Dunnet’s multiple comparison test); (**b**) Effects of pharmacological inhibition of NRF2 with Brusatol (10 nM, added for the entire differentiation culture period) on OC formation, assessed by TRAP staining. Quantification of the number of OCs per well (mean ± SD, n = 9 independent experiments, Welch’s *t*-test); (**c**) Effects of the combined inhibition of NRF2 (Brusatol, 10 nM) and UCHL1 (LDN57444, 10 nM) on OC formation, assessed by TRAP staining. Left, representative images, right, quantification of the number of OCs per well (mean ± SD, n = 9 independent experiments, paired *t*-test; (**d**) Analysis of intracellular ROS upon pharmacological UCHL1 inhibition assessed by CellROX staining (MFI ± SD, n = 3 independent experiments, *t*-test); (**e**) Representative images of immunoblot analyses of KEAP1 and HMOX1 protein levels in preOCs treated for 24 h with LDN57444 (20 µM) and/or ascorbic acid (30 ng/mL); (**f**) Effects of the combined treatment with ascorbic acid and LDN57444 at the indicated doses on OC formation, assessed by TRAP staining. Quantification of the number of OCs per well (mean ± SD, RM two-way ANOVA with Dunnet’s multiple comparison test); (**g**) Immunoblot analysis of KEAP1 and HMOX1 upon LDN57444 (20 µM) or vehicle treatment for 24 h. Left, representative images; Right, quantified band intensities normalized on actin (mean ± SD, n = 5 and 3 independent experiments respectively, Welch’s *t*-test, *p < 0.05, **p < 0.01); (**h**) qRT-PCR of KEAP1 and HMOX1 mRNAs upon LDN57444 (20 µM) or vehicle treatment for 24 h (mean ± SD, n = 4 independent experiments, Welch’s *t*-test); (**i**) Representative images of immunoblot analysis of KEAP1 and HMOX1 upon LDN57444 and/or bafilomycin-A1 (Baf-A1, 20 nM 24 h) treatment.

NRF2 activity has been reported to suppress OC differentiation, as increasing or silencing *Nrf2* caused impaired or enhanced in vitro OCgenesis, respectively^41^. To test if NRF2 is involved in the essential role played by UCHL1 in OC differentiation, we first checked the dynamics of NRF2 activity and observed a reduction of NRF2 and HMOX1 protein abundance along unperturbed OCgenesis (Fig. 6A). To establish if enhanced NRF2 activity is causally involved in the mechanism whereby UCHL1 blockade suppresses OC differentiation, we antagonized NRF2 with the commercial inhibitor brusatol^42,43^. NRF2 inhibition increased OC formation from wild-type BMMs, confirming the previously reported inhibitory role of NRF2 activity in OCgenesis (Fig. 6B). Moreover, preventing NRF2 activation partially rescued the suppression of OC differentiation mediated by UCHL1 inhibition (Fig. 6C), causally implicating heightened NRF2 activity in the observed blockade of OCgenesis. We then investigated the mechanism whereby UCHL1 inhibition stimulates NRF2. First, we tested the role of oxidative stress, the prime trigger of NRF2 activity. We found that ROS intracellular levels were not altered upon LDN57444 treatment (Fig. 6D). Moreover, treatment with ascorbic acid, an established antioxidant, failed to dampen NRF2 activation, as attested by unabated protein abundance of the prototypical NRF2 target, HMOX1 (Fig. 6E). These results indicate that NRF2 activation following UCHL1 inhibition is not mediated by increased oxidative stress. Furthermore, OCgenic assays employing different concentrations of UCHL1 inhibitor and ascorbic acid demonstrated that reducing oxidative stress has no impact on the anti-OCgenic effect of UCHL1 inhibition (Fig. 6F), conclusively excluding redox homeostasis from the mechanism whereby UCHL1 controls NRF2 activity and OCgenesis. However, we noted that UCHL1 inhibition significantly reduced the abundance of KEAP1, the adapter that suppresses NRF2 activity by promoting its ubiquitination and subsequent proteasomal degradation^39^ (Fig. 6G). Although NRF2 suppression by KEAP1 is known to be tuned by oxidative stress^39^, the negative impact of UCHL1 inhibition on KEAP1 was redox-independent (Fig. 6E), suggesting an alternative regulatory mechanism. KEAP1 is known to be constitutively degraded by autophagy^44^. We then hypothesized a specific inhibitory regulation of NRF2 exerted by UCHL1 in OCgenic precursors by sustaining the protein abundance of KEAP1. In keeping with this hypothesis, we found that UCHL1 inhibition decreases KEAP1 protein abundance without affecting its mRNA expression (Fig. 6G-H). Accordingly, unlike redox modulation by ascorbic acid, autophagic blockade prevented UCHL1 inhibition-induced NRF2 activation, as revealed by HMOX1 expression levels (Fig. 6I). Altogether, our findings implicate NRF2 activity in the mechanism whereby UCHL1 supports OC differentiation, through a novel mechanism driven by oxidative stress-independent stabilization of KEAP1.

## Discussion

Our study originates from the idea that a comprehensive proteomic characterization of primary OCgenesis could disclose new relevant and actionable regulatory circuits. Indeed, the molecular phenotyping of OC differentiation available in the literature relies mainly on RAW264.7, a murine leukemic cell line, as monocytic precursors. While this model offers a valuable reductionist surrogate of OC progenitors, substantial differences exist^45^, as exemplified by deregulated, M-CSF-independent proliferation of transformed monocytes, which limit its value and prompt to employ primary BMMs to investigate physiologic OCgenesis. Moreover, few comprehensive phenotypic studies of OCs have been conducted through proteomics^45,46^, while most rely exclusively on transcriptomic profiling^47–49^. We thus reasoned that a thorough characterization of the proteome phenotypic layer assessed dynamically at different stages of OC differentiation would add valuable insight. To this end, we deployed a twofold proteomic strategy, employing label-free and SILAC proteomics, which successfully and concordantly defined comparable protein profiles, with most representative gene products identified in both analyses, revealing a novel protein signature associated with OCgenesis.

The unsupervised separation of BMMs, preOCs, and mature OCs demonstrated that proteomics captured protein changes relevant to OC ontogenesis (Fig. 1). Moreover, our study consolidated established OC-associated biological features, such as reshaping of the lysosomal compartment, with OC-specific components accumulating within an otherwise not expanded organellar proteome, consistent with its key role in bone resorption, validating our approach. The most notable change, by number of proteins involved, related to the mitochondrial proteome, which appeared significantly increased along OCgenesis (Fig. 2), consistent with previous reports of high mitochondrial abundance and respiratory activity in models of OC differentiation from RAW264.7 or human primary precursors^50–52^. Interestingly, our analysis revealed that the progressive increase of the mitochondrial proteome during OCgenesis is largely accounted for by protein components of OXPHOS and the TCA cycle, in keeping with the superior energy demand arguably imposed by bone resorption.

The proteomic signature of OCgenesis described herein also offers a valuable framework for prospective studies aimed to dissect the molecular circuits regulating OC differentiation. Indeed, most signature proteins identified are not currently associated with OC biology according to the GO database (Fig. 1,2), reflecting the current knowledge of OC ontogeny based on transcriptomic, but not proteomic profiling. Indeed, of the top 500 DEPs identified in OCs (vs BMMs) in our study, only 8 are also associated in GO to OC differentiation (GO:0030316). Similarly, only 2 genes that we found most deregulated from the early phases of differentiation were already present in the same database. Thus, our newly defined signature may help fill a knowledge gap and inform further molecular and functional investigations. Moreover, analysis of the identified OC-associated DEPs in GO databases showed that only 5% of the genes are currently included in bone-related, and only 3% in OC-related, pathways. Nevertheless, ~ 20% of OC DEPs are associated to skeletal phenotypes in the MGI database, supporting their ultimate relevance to bone biology. Most interestingly, ~ 15% of DEP-coding genes are not annotated in the MGI database and, given the strong correlation with OC differentiation in our unbiased analysis, hold potential relevance to OC biology and bone pathophysiology. Arguably, the skeletal impact of genes might have escaped investigators due to general lethality (~ 2.8% of genes), emergence of more prominent or confounding phenotypes (~ 44.8% were annotated for non-skeletal phenotypes), omitted skeletal phenotyping, functional adaptations, and strain/species-specific characteristics. Overall, most OC DEPs reported herein do not show extensive characterization in the available databases.

The prime goal of our proteomic characterization of OCgenesis was to identify new OCgenic players and potential therapeutic targets, as epitomized by UCHL1, a protein with virtually no known function in bone, which stood out as the most upregulated gene product in the cytosol of differentiating OCs (Fig. 3). UCHL1 is a member of the UCHL family of Ub deconjugating enzymes (DUB), with a recently proposed, yet controversial additional activity as a Ub ligase (E3)^53^. UCHL1 has predominantly been studied in the neurological and cardiovascular fields, especially for its role in tissue repair, and in oncology, with both pro- and anti-tumoral cancer-specific functions described. In humans, genetic *Uchl1* alterations have been associated to neurological disorders, e.g., spastic paraplegia and Parkinson’s disease, mainly due to altered proteostasis via accumulation of ubiquitinated proteins and/or impaired autophagic clearance^31–37^.

The scarce mentions of UCHL1 in OC or bone biology prompted us to investigate it further. *Uchl1* null mice develop severe axonal disease (gracile axonal dystrophy) due to neuromuscular junction abnormalities and die within few months^31^. They were also reported to have reduced bone mass, a typical phenotypic correlate of severe locomotor diseases^54^. In view of our findings and of the previously reported high *Uchl1* expression in patients with autosomal dominant osteopetrosis type II^29^, we investigated the role of UCHL1 in OC differentiation. First, we observed that UCHL1 expression starts to rise early in differentiating OCs, preceding that of prototypical OC-associated factors such as *Acp5* and *Ctsk*, suggesting an upstream pro-OCgenic role. Indeed, blocking its expression or activity respectively through genetic or pharmacological inhibition potently suppressed OC formation (Fig. 3).

OC differentiation has been shown to rely on the UPR and to be suppressed by proteasome inhibition, a treatment known to profoundly perturb protein homeostasis, inducing accumulation of unfolded and misfolded proteins in the cytosol and in the ER^38,55^. Given UCHL1’s proposed function as a multi-target DUB maintaining Ub availability and cellular proteostasis, we hypothesized that UCHL1 inhibition could impair OC differentiation through accumulation of undigested ubiquitinated proteins and/or impairment of autophagy. However, we did not observe any accumulation of Ub-conjugated proteins upon UCHL1 inhibition, nor any impairment of global autophagic flux (Fig. 4), ruling out a pan-DUB function maintaining OC proteostasis. We thus hypothesized an alternative pro-OCgenic mechanism exerted through the control of specific targets.

Recently, a negative role of UCHL1 was proposed in OC differentiation, exerted through downregulated NFATc1 signalling. In this work, loss of UCHL1 in myeloid cells did not change bone mass or OC formation in vivo, whereas a pro-inflammatory challenge resulted in enhanced formation and activity of OCs^56^. These findings are in striking contrast with our study that unequivocally demonstrates that UCHL1 is essential for OC differentiation. A possible explanation, the apparent discrepancy could in fact depend on diverse differentiation protocols and inflammatory stimuli which may drastically change macrophage selection, polarization, and potential to undergo OCgenesis^19^. Further studies are warranted to challenge the role of UCHL1 in pathologic and inflammatory conditions.

To unbiasedly identify proteins regulated by UCHL1 that account for its essential pro-OCgenic function, we then deployed a comprehensive proteomic analysis on preOCs following genetic or pharmacologic UCHL1 inhibition. This approach led us to identify, together with the expected downregulation of OCgenesis, a clear upregulation of NRF2 activity, which we confirmed by dedicated targeted qRT-PCR and immunoblotting experiments (Fig. 5). The transcriptional activator NRF2 mediates the main antioxidant cellular pathway, initiating with cytosolic stabilization of NRF2, followed by its nuclear relocalization and transactivation of a vast array of antioxidant genes involved in NADPH production, glutathione synthesis, cellular detoxification, iron homeostasis, and pentose phosphate pathways^39^.

The role of oxidative stress in health and disease is complex and somewhat controversial, since ROS can be both beneficial and detrimental depending on their levels, cell type, metabolic activity, and microenvironmental cues^41^. In vitro studies have consistently reported an anti-OCgenic role of NRF2 activity^57–62^, whose enhancement or inhibition respectively impairs or promotes OC formation, as confirmed in our ex vivo model of OCgenesis from primary mouse BMMs (Fig. 6). The anti-OCgenic effect of NRF2 activity has often been imputed to its canonical antioxidant function, in view of the proposed essential roles of ROS in OCgenic signalling pathways (e.g., NF-kB, AKT, MAPK). The anti-OCgenic effects of ROS have also been confirmed in vivo, although genetic NRF2 inactivation had profound effects both on OCs and osteoblasts, two effector cell types with substantial reciprocal interplay, raising complexity^57,63,64^.

The rationale linking UCHL1 to NRF2 is manifold. A functional NRF2 antioxidant response is essential to prevent the pathogenetic effects of oxidative stress in neurodegeneration and tissue damage, conditions in which UCHL1 has been implicated^23,25,65^. Of note, mutant UCHL1 was found to reduce ROS and protect against oxidative stress in Parkinson’s disease^66^, although the underlying molecular mechanism remained undetermined. Moreover, UCHL1 inhibition was shown to ameliorate retinal degeneration via reduced oxidative stress^67^. Finally, the *Uchl1* gene was proposed as a new target of NRF2, while UCHL1 protein function was reported to be vulnerable to oxidation, hence acting as a ROS sensor under high oxidative stress^68,69^.

Building on this background and on our observed impact of UCHL1 inhibition on NRF2 activity, we therefore hypothesized that the magnification of NRF2 response might account for UCHL1 blockade-driven suppression of OCgenesis. In keeping with this hypothesis, preventing NRF2 activation was sufficient to restore, in part, OC formation, contrasting the effects of UCHL1 inhibition (Fig. 6).

Having established a causal role of NRF2 in the previously unrecognized essential role of UCHL1 in OCgenesis, we then set out to define the underlying mechanism. Our data demonstrate that the control of NRF2 by UCHL1 is not exerted through increased oxidative stress, as revealed by unchanged levels of ROS and comparable OCgenesis impairment upon UCHL1 inhibition in the presence of an antioxidant. Rather, the data suggest that UCHL1 curbs NRF2 signalling through increased stability of KEAP1, the well-known adapter controlling NRF2 degradation^39,44^. Indeed, following UCHL1 inhibition, KEAP1 protein abundance was rapidly reduced, even in the presence of antioxidants, while its mRNA levels remained unchanged. Of note, heightened NRF2 activity upon UCHL1 blockade was rescued by concomitant lysosomal inhibition, further implicating KEAP1 stabilization in UCHL1-dependent control of OCgenesis (Fig. 6).

Altogether, the data reveal a novel regulation of NRF2 signaling and raise the intriguing possibility that UCHL1 may act as a previously unrecognized direct deubiquitinase of KEAP1. Besides the herein demonstrated role of this new axis in the control of OCgenesis, considering the importance of NRF2 activity, the significance of the novel regulatory role played by UCHL1 may extend beyond OC biology.

In conclusion, our work defines a new proteomic signature of murine OCgenesis that may inform further studies on the molecular phenotype of OCs and on the circuits controlling their differentiation. Moreover, our unbiased approach identifies UCHL1 as an unprecedented essential driver of OC formation, acting through a novel KEAP1-mediated inhibitory control of NRF2. The relevance of UCHL1 in human bone biology and its involvement in skeletal diseases warrant further investigations, particularly across the heterogeneous ontogeny of OCs, and in view of its regulation of the activity of NRF2, a signalling hub interfacing different critical pathways in health and disease. Our report may encourage prospective studies aimed to further elucidate additional molecular mechanisms regulating the newly identified UCHL1/KEAP1/NRF2 axis, as well as to gauge its relevance under physiologic and pathologic conditions in vivo.

## Materials and methods

### Osteoclastogenesis, cell cultures, and drug treatments

All mouse experiments presented in this study were reviewed and approved by the Institutional Animal Care and Use Committee, IRCCS Ospedale San Raffaele, Milano, Italy (protocol #1163) and authorized by the Italian Ministry of Health (protocol #451/2021-PR). All mouse studies were then conducted in accordance with the approved protocols and conformed with the Italian guidelines and regulations (D.lgs. 26/2014) and are reported in accordance with the ARRIVE guidelines (https://arriveguidelines.org). Male and female adult (3–6 months-old) C57Bl/6 wild-type mice (Charles River) were euthanized via CO_2_ asphyxiation. Next, hind limbs were excised, muscle and connective tissues carefully removed, and femurs and tibiae isolated. Proximal tibial and distal femoral epiphyses were cut to access the bone marrow, and invertedly inserted in a 0.5 mL tube perforated at the bottom, inside a 1.5 mL tube. The bone marrow was then collected by rapid centrifugation (30 s at 10,000 *g*) and resuspended in complete medium (alpha-MEM, Gibco-Life Technologies, 22571-038) supplemented with 10% FBS (Hyclone, SH30070.02), penicillin/streptomycin (100 U/mL; Gibco-Life Technologies, 15140-122), and glutamine (2 mM; Gibco-Life Technologies, 25030-024). Cells were then strained through a 70 µm filter, centrifuged, and plated in petri dishes in complete alpha-MEM with 100 ng/mL M-CSF (Peprotech, 315–02) to select bone marrow derived monocytes (BMMs). After 48 h the supernatant was removed, BMMs washed twice in PBS and re-incubated with medium and M-CSF for expansion for 72 h. BMMs were then washed in PBS, detached in trypsin EDTA (Gibco-Life technologies, 15,400–054) and plated for OCgenesis in complete alpha-MEM with 10 ng/mL M-CSF and 100 ng/mL RANKL (BioLegend, 577102). Medium was changed every two days until OC formation (7 days). TRAP staining of OC was performed with the Leukocyte Acid Phosphatase (TRAP) Kit (Sigma-Aldrich, 387A-1KT), according to manufacturer’s instructions, after removing the medium, fixing the cells in formalin from 10’ and washing twice in PBS. OCs were identified as TRAP positive cells presenting at least 3 nuclei. Cells were treated as indicated in the figure legends with: 20 nM bafilomycin-A1 (Cayman Chemical, 11,038), LDN57444 (Sigma-Aldrich, L4170), 1 µM bortezomib (Cell Signaling, 2204), 50 µM anisomycin (Sigma-Aldrich, A9789), 10 nM brusatol (Sigma-Aldrich, SML1868) and ascorbic acid (Sigma-Aldrich, A7506).

### Genetic manipulation

Lentiviral viruses to stably express anti-*Uchl1* and control shRNAs were generated starting from Mission shRNAs (Sigma-Aldrich, non-target shRNA: SHC002; sh*Uchl1* (sh1): TRCN0000008443; sh*Uchl1* (sh2): TRCN0000008444) were cloned in a pLKO.5 plasmid homemade modified to replace the expression of puromycin resistance with tGFP. Lentiviral vectors were packaged with shRNAs, pMD2-VSV-G, pMDLg/pRRE and pCMV-Rev plasmids in HEK 293 T cells for 14 h in IMDM (Gibco-Life Technologies 12440-053) supplemented with 10% FBS (Hyclone, SH30070.02), penicillin/streptomycin (100 U/mL; Gibco-Life Technologies, 15140-122), and glutamine (2 mM; Gibco-Life Technologies, 25030-024), then medium was replaced. 30 h after medium change cell supernatants were collected, ultra-centrifuged, filtered and added to BMM for 16 h. OCgenesis was induced 48 h after infection and UCHL1 silencing was verified both prior and after OCgenesis induction in BMMs and preOCs, respectively.

### Immunoblotting

Total cellular extracts were obtained by lysing cells in 150 mM NaCl, 10 mM Tris–HCl (pH 7.5), protease inhibitor cocktail (Roche, 05056489001) and 1% SDS (Sigma-Aldrich, 05030). Genomic DNA was mechanically removed using 0,5 mL Insumed syringes (PIC solution) or sonication. Protein concentration was measured using Bio-Rad DC protein assay following manufacturer instructions. Western blots were performed using 10–30 µg of protein lysate in homemade (8–15%) SDS-PAGE gels, or 4–12% Bolt pre-casted gels (Thermo Fisher, NW04120). Images were obtained using Uvitec Imager Mini HD9 (Uvitec Ltd) for HRP-conjugated secondary Ab or FLA9000 (FujiFilm) for Alexa Fluor conjugated secondary antibodies. Densitometric analysis was performed using ImageJ software (http://rsbweb.nih.gov/ij/). Full-length images are included in Supplementary Material. Antibodies used were: mouse anti-β-Actin (Sigma-Aldrich, A5441); rabbit anti-UCHL1 (Genetex, GTX32782); rabbit anti-p62 (Sigma-Aldrich, P0067); rabbit anti-HMOX1 (Abcam. Ab68477); rabbit anti-PRDX1 (AbFrontier, LF-MA0095); rabbit anti-ACP5/TRAP (Genetex, GTX30018); rabbit anti-CTSK (Genetex, GTX59712); rabbit anti-NRF2 (Proteintech, 16396-1-AP); rabbit anti-Aconitase2 (kind gift of Dr. Paolo Santambrogio, Milan, Italy), mouse anti-SDHA (MitoSciences, MS204), rabbit anti-cleaved caspase-3 (Cell Signaling 9664); rabbit anti-KEAP1 (Proteintech, 10503-2-AP), rabbit anti-LC3A (Novus Biological, NB100-2331), mouse anti-ubiquitin (Santa Cruz, P4D1 clone, sc-8017), Alexa Fluor 647 goat anti-mouse IgG (Life Technologies, A21236); Alexa Fluor 647 goat anti-rabbit IgG (Life Technologies, A21245); Anti-rabbit IgG, HRP-linked (Southern Biotech, 4050-05); Anti-mouse IgG, HRP-linked (Southern Biotech, 1031-05).

### Quantitative RT-PCR

RNA was extracted with TriFAST (Euroclone, EMR507100), up to 1000 ng RNA retro-transcribed with ImProm-II Reverse Transcriptase System (Promega, A3800), and cDNA corresponding to 5 ng of original RNA used as template in qPCR reactions. qPCRs were performed using SYBR green I master mix (Roche, 04887352001) on Roche LightCycler 480 or iTaq SYBR Green Supermix (Bio-Rad, 1725122) on Bio-Rad CFX96 PCR. Data were analyzed on Roche LC480 software using Advance Relative Quantification or on Bio-Rad CFX Maestro.

Primers used were:

**Table:** 

Gene	Primers
*β-actin*	CCGCGAGCACAGCTTCTTTG AGTCCTTCTGACCCATTCCCAC
*Uchl1*	TGGTACCATCGGGTTGATCC TGGTTCACTGGAAAGGGCAT
*Acp5*	CAAAGAGATCGCCAGAACCG GAGACGTTGCCAAGGTGATC
*Ctsk*	CATCTTTGGAGTGAGCACCA GCATCCAAAACAGCCATCTTA
*Me1*	AGAGCAGTGCTACAAGGTGACC CCAAGAGCAACTCCAGGGAACA
*Sqstm1*	AGAATGTGGGGGAGAGTGTG TCTGGGGTAGTGGGTGTCAG
*Hmox1*	ACGCATATACCCGCTACCTG AAGGCGGTCTTAGCCTCTTC
*Gclm*	TCCTGCTGTGTGATGCCACCAG GCTTCCTGGAAACTTGCCTCAG
*Keap1*	GATGGCCACATCTACGCAGT ATCCTCCGTGTCAACATTGG

### Flow cytometric analyses

For total ROS analysis cells were washed in PBS and detached with trypsin EDTA (Gibco-Life technologies, 15400-054), incubated for 5 min, collected in complete alpha-MEM medium and stained with CellROX Deep Red 5 µM (Life Technologies, C10422) for 20 min at 37 °C. Cells were then washed twice in cold PBS and analysed by FACS. Data were obtained with Cytoflex S Flow Cytometer (Beckman Coulter) and analyzed using the FlowJo Software.

### MTT assay

BMMs, obtained as descried above, were plated in triplicate at a concentration of 1 × 10^4^ cells/well in 96-well plates. The cells were incubated overnight in humidified air with 5% CO_2_ at 37 °C. They were subsequently treated with serial dilutions of DMSO or LDN57444 for 48 h. At the end of treatment, Thiazolyl Blue Tetrazolium Bromide solution (0.5 mg/mL final concentration; Sigma-Aldrich, MM5655) were added to the culture medium and incubated for 4 h at 37 °C. After culture medium removal, the formazan crystals were dissolved by adding 100 μl DMSO (Sigma-Aldrich, D2650), and the plate was read at 570 nm, as the reference wavelength, using a microplate reader (Model 680; Bio-Rad Laboratories Inc).

### Macrophage polarization

BMMs, obtained as previously described, were induced to differentiate towards M1-type macrophages, M2-type macrophages or osteoclasts, through stimulation of IFN-γ (50 ng/mL, PeproTech, 315–05), IL-4 (20 ng/mL, Miltenyi Biotec, 130–094-061) and M-CSF (10 ng/mL), or M-CSF (10 ng/mL) and RANKL (100 ng/mL), respectively, for 48 h in complete alpha-MEM, as previously described.

### Proteomics

*Label-free proteomics* Label-free proteomics of primary OCgenesis was performed on six 4 months-old C57BL/6 mice (3 females and 3 males). OCgenesis was carried our as described above. Cells were collected for protein isolation at different time points of differentiation as BMMs, preOCs and OCs (day 0, 3 and 7 of differentiation, respectively) as described in the immunoblot section above. 30 µg of protein lysate underwent processing, digestion, isolation as previously described in Fucci et al.^70^ and tryptic peptides were analyzed by LC–MS/MS with a Q-Exactive mass Spectrometer (Thermo Fisher Scientific) as described in^71^. Raw data were processed with MaxQuant (version 1.6.1.0) and peptides identified from MS/MS spectra against the Mouse Uniprot Complete Proteome Set database (2022_05 for label-free OCgenesis, 2019_02 for *Uchl1* silencing, 2021_06 for LDN57444) using the Andromeda search engine. Imputation and statistical analyses were performed on Perseus Software (v 2.0.3). The proteins identified (3171) were filtered for potential contaminant and those with valid LFQ intensities (> 0) in less than 6/18 samples were excluded. Missing values were then imputed by replacing from gaussian distribution, with a resulting working list of 2238 genes. Heatmaps and clustering analyses were generated using Broad Institute Morpheus Software (https://software.broadinstitute.org/morpheus), showing relative values for each row. Hierarchical Clustering was performed with One minus Pearson correlation (linkage = average; cluster = columns and rows) and k_means clustering with 3 expected clusters (max number of iterations = 10).

Statistically significant (one way ANOVA test) deregulated genes were 1119 in OCs versus BMMs and 617 in preOCs versus BMMs.

Pathway enrichment analysis was performed on the top 250 [representing ~ 20% edge] upregulated or downregulated proteins in OCs vs BMMs. Enrichment analyss was performed in Cytoscape (2.5.8) ClueGO (3.9.1) in databases of Gene Ontology Biological Processes, Cellular Component and Molecular Function (EBI-UniProt-GOA-ACAP-ARAP-13.5.2021), and KEGG-13.5.2021 pathways. The following analysis parameters were adopted: Enrichment (Right-sided hypergeometric test);* p* value correction by Benjamini–Hochberg < 0.05; Min GO Level = 5; Max GO Level = 10; Number of Genes = 6; Min Pathway Coverage = 20.0%; Custom Reference Set = 2238 genes. To represent the resulting upregulated (240) or downregulated pathways (170), organized in 40 or 50 groups/clusters, respectively, the pathway term with the highest *p* value for each group was selected (Fig. 1D, Suppl. Figure 1; full list in Suppl. Datasets 1 and 2). The dataset of OCgenic signature comprises the top100 proteins that were deregulated in both preOC and OC samples, compared to BMM (Fig. 2B, Suppl.Dataset 1). Statistically significant (Two-way ANOVA) sex-dependent genes in OCgenesis (246 in preOCs vs. BMMs and 145 in OCs vs. BMMs) underwent pathway analysis as described above.

Label free proteomics on UCHL1-inhibited/silenced OCgenesis was performed on preOCs (72 h RANKL-induced OC differentiation) treated with 20 µM of LDN57444 and BMMs and preOCs silenced for *Uchl1* (sh1, sh2) as described above. Processing of proteins and mass spec analysis previously described. Proteins that were differentially expressed in both LDN57444 vs DMSO and sh*Uchl1* vs shMock (135 DEP; consistently deregulated in at least 4/5 replicates and with at least 25% mean differential expression) were taken into consideration for gene enrichment analysis. DEPs (93 upregulated and 41 downregulated) were analyzed with ClueGO for pathways enrichment, (min 10% coverage, min 3 genes) as previousy described above.

*SILAC proteomics* For SILAC analysis BMM were grown in complete SILAC RPMI medium (Thermo Scientific, 88,365) supplemented with 50 µg/mL ascorbic acid and ‘‘light’’ (Lys0, Arg0; Cambridge Isotope Laboratories, ULM-8766 and ULM-8347) or ‘‘heavy’’ (Lys8, Arg10; Cambridge Isotope Laboratories, CNLM-291 and CNLM-539-H) amino acids to collect BMMs and OCs, respectively. Labeled BMMs were differentiated in OCs and lysed as described above. To prepare samples for MS/MS analysis, 60 µg of total proteins from mixed cellular extracts were loaded on Amicon Ultra- 0.5 mL 10 K (Millipore, UFC501096) and washed thoroughly with 8 M Urea in 0,1 M Tris HCl pH 8 through centrifugation. Proteins were processed and digested as described for label-free analyses, then, peptides were analysed by LC–MS/MS. Raw data were processed with MaxQuant (version 1.5.2.8). The peptides and protein false discovery rates (FDR) were set to 0.01; minimum two peptides and at least one unique peptide were required for high-confidence protein identification. The statistical program Perseus (v.1.5.1.6.) was used for determining the Significance B with a *P* value < 0.05%.

Proteins were run through functional annotation clustering on DAVID web resource, using GOterm-CC-DIRECT, GOterm BP-DIRECT and KEGG pathways as search parameters. Cell compartments showed in Fig. 2C and/or Suppl. Figure 4A represent following categories: GO:0005829 ~ cytosol, GO:0005634 ~ nucleus, GO:0005739 ~ mitochondrion, GO:0005783 ~ endoplasmic reticulum, GO:0005856 ~ cytoskeleton, GO:0005794 ~ Golgi apparatus, GO:0005840 ~ ribosome, GO:0005743 ~ mitochondrial inner membrane, GO:0009986 ~ cell surface, GO:0005764 ~ lysosome, KEGG mmu00190:Oxidative phosphorylation, GO:0006099 ~ tricarboxylic acid cycle, GO:0061621 ~ canonical glycolysis, GO:0045453 bone resorption.

### Gene-Phenotype annotation

The top 500 DEPs in OCs obtained from label-free proteomics were analysed for mammalian phenotype correlations and gene ontology annotation through the Mouse Genome Informatics (MGI) database^22^. Skeletal phenotypes include entries such as “bone deformities, skeletal diseases, increased/decreased bone mass, osteoclast/osteoblast abnormalities etc”.

### Statistical analyses

Graphs and data analysis were obtained using Prism v10.0.3 software (GraphPad). Statistical significance was tested as indicated in the figure legends. Asterisks indicate the following *p* values: *p < 0.05; **p < 0.01; ***p < 0.001; ****p < 0.0001. Figures were assembled with Adobe Illustrator.

### Supplementary Information

Supplementary Information 1.

Supplementary Information 2.

Supplementary Information 3.

Supplementary Information 4.

Supplementary Figures.

## Data Availability

All the data that support the findings of this study are provided within the manuscript and supplementary information files.
