# Supplementary material for: A novel proteomic signature of osteoclast differentiation unveils the deubiquitinase UCHL1 as a necessary osteoclastogenic driver
Source: Sci Rep. 2024 Mar 27;14:7290. doi: 10.1038/s41598-024-57898-y (PMC10973525; doi:10.1038/s41598-024-57898-y)
Supplement: Supplementary file 5 — Supplementary Figures. [file 41598_2024_57898_MOESM5_ESM.pdf]

Supplementary Information

A Novel Proteomic Signature of Osteoclast Differentiation Unveils the Deubiquitinase UCHL1 as a Necessary Osteoclastogenic Driver

Maria Materozzi[1,2], Massimo Resnati[1], Cecilia Facchi[1,2], Matteo Trudu[1,2], Ugo Orfanelli[1], Tommaso Perini[1,2], Luigi Gennari[3], Enrico Milan[1,2,4,5,*], and Simone Cenci[1,2,4,5,*]

1. Age Related Diseases Unit, IRCCS Ospedale San Raffaele, Milano (Italy);
2. Università Vita-Salute San Raffaele, Milano (Italy);
3. Department of Medicine, Surgery and Neurosciences, University of Siena, Siena (Italy).
4. Senior authors
5. These authors contributed equally

* Correspondence: milan.enrico@hsr.it (E.M.), cenci.simone@hsr.it (S.C.)

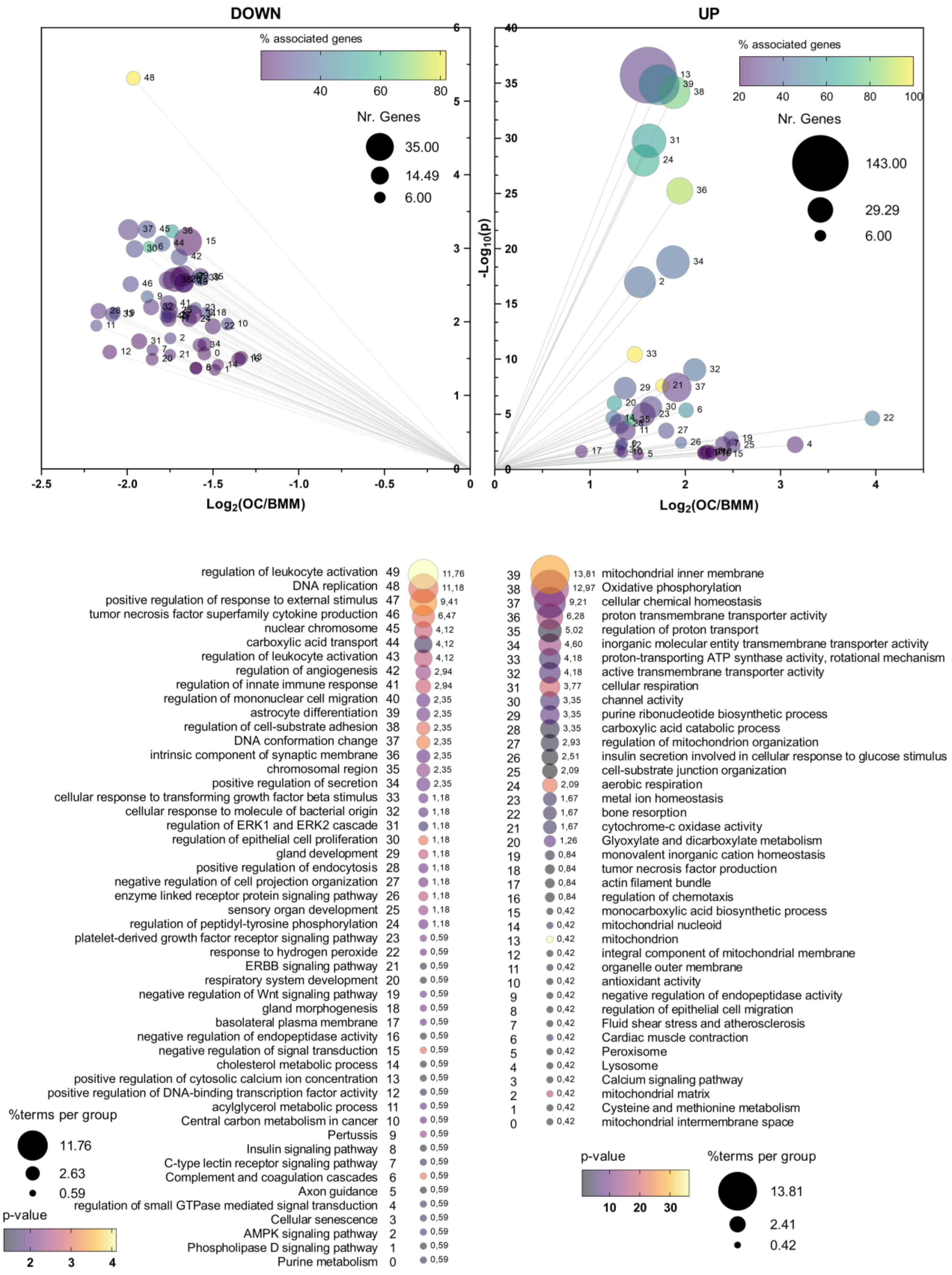

Supplementary Figure 1.

Gene ontology (GO) enrichment analysis of the OC proteome compared to BMM by ClueGO showing enriched (right) and depleted (left) pathways clustered in 40 and 50 groups, respectively. Above: representative term per group; below: list of groups with relative terms composition (%) and p-value (-Log_{10}).

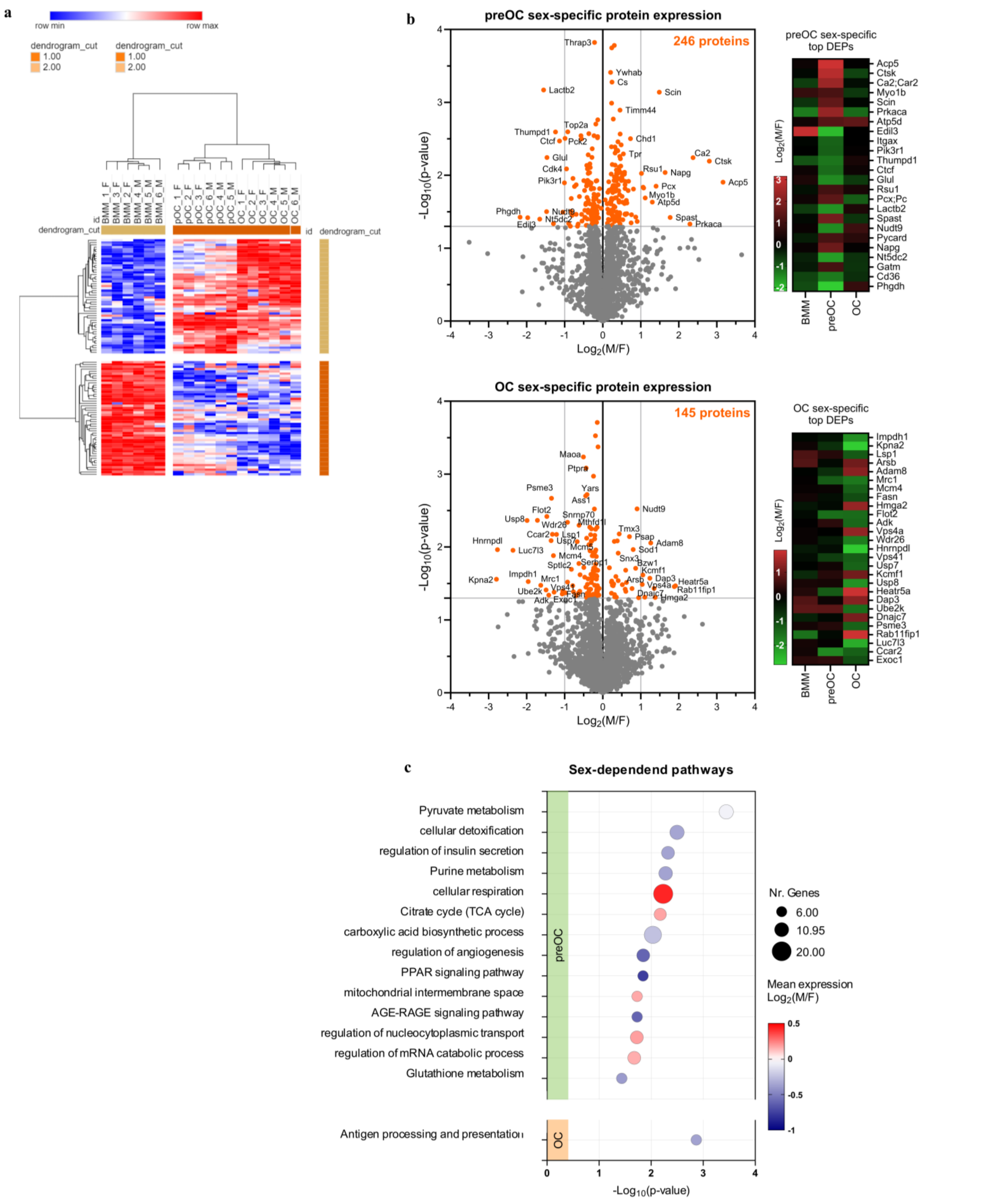

Supplementary Figure 2.

a) Hierarchical and k_means clustering heatmap of label-free MS/MS proteomics showing DEPs consistently deregulated in preOCs (pOCs) and OCs; **b)** Proteins (gene names) deregulated in a sex-dependent manner in preOCs (top) and OCs (bottom); left, volcano plots; right, heatmaps showing the highest differences (log_2 of male to female mean ratio) and their expression in BMMs, preOCs, and OCs; **c)** Gene ontology (GO) enrichment analysis of the sex-dependent DEPs in preOCs or OCs.

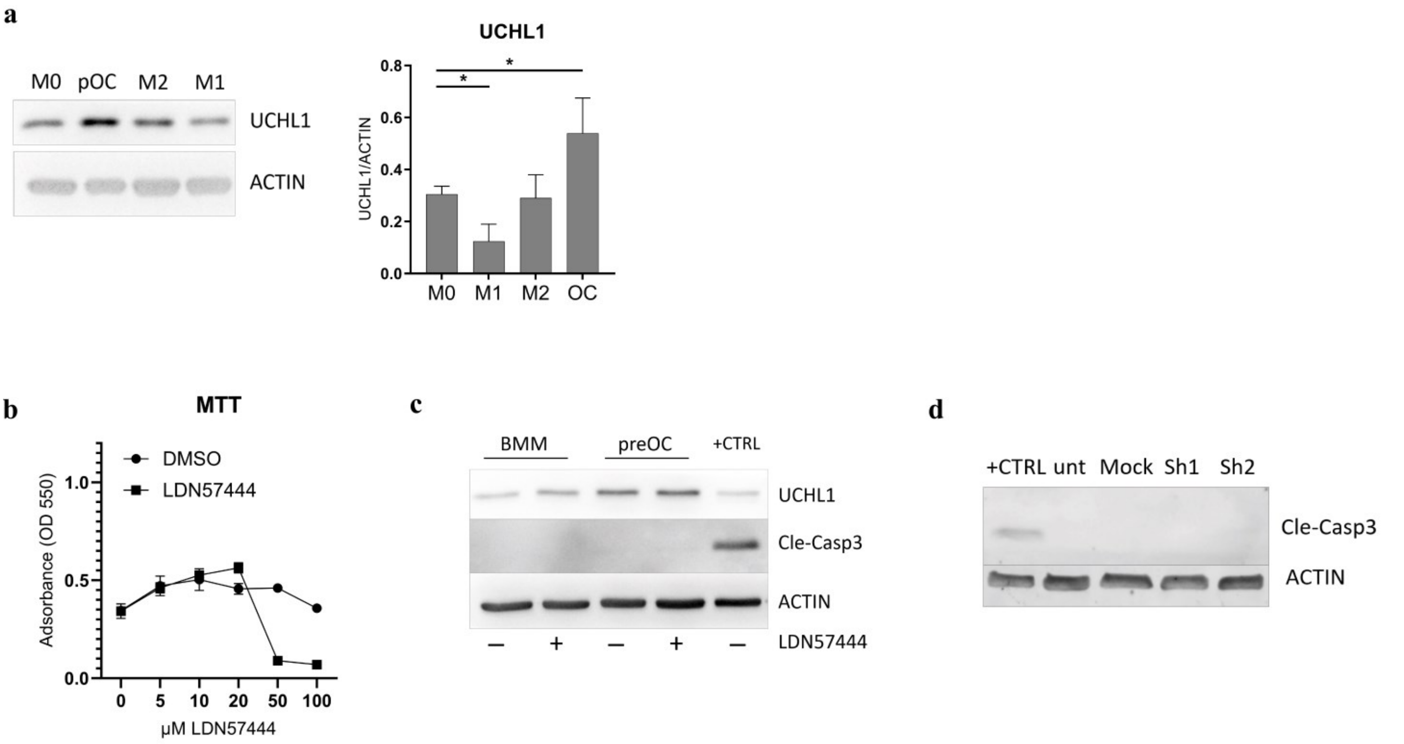

Supplementary Figure 3.

a) Immunoblot analysis of UCHL1 in BMMs undergoing macrophage polarization (M1, M2) or OC differentiation; left, representative images; right, quantifications of band intensities normalized on actin (mean ± SD, n=3 independent experiments, One-way ANOVA with Dunnet multiple comparison test vs BMMs); **b)** MTT viability assay in BMMs treated with the indicated doses of LDN57444 or DMSO for 48h (representative curve of 1 of 3 independent experiments); **c)** Representative immunoblots of UCHL1 and cleaved caspase 3 in LDN5744-treated (20 µM, 24 h) BMMs and preOCs; BMMs treated with anisomycin (50 µM, 6 h) were used as a positive control of apoptosis activation; **d)** Representative immunoblots of cleaved caspase 3 in UCHL1-silenced preOCs. BMMs treated with anisomycin (50 µM, 6 h) were used as a positive apoptotic control as in (c).

a

b

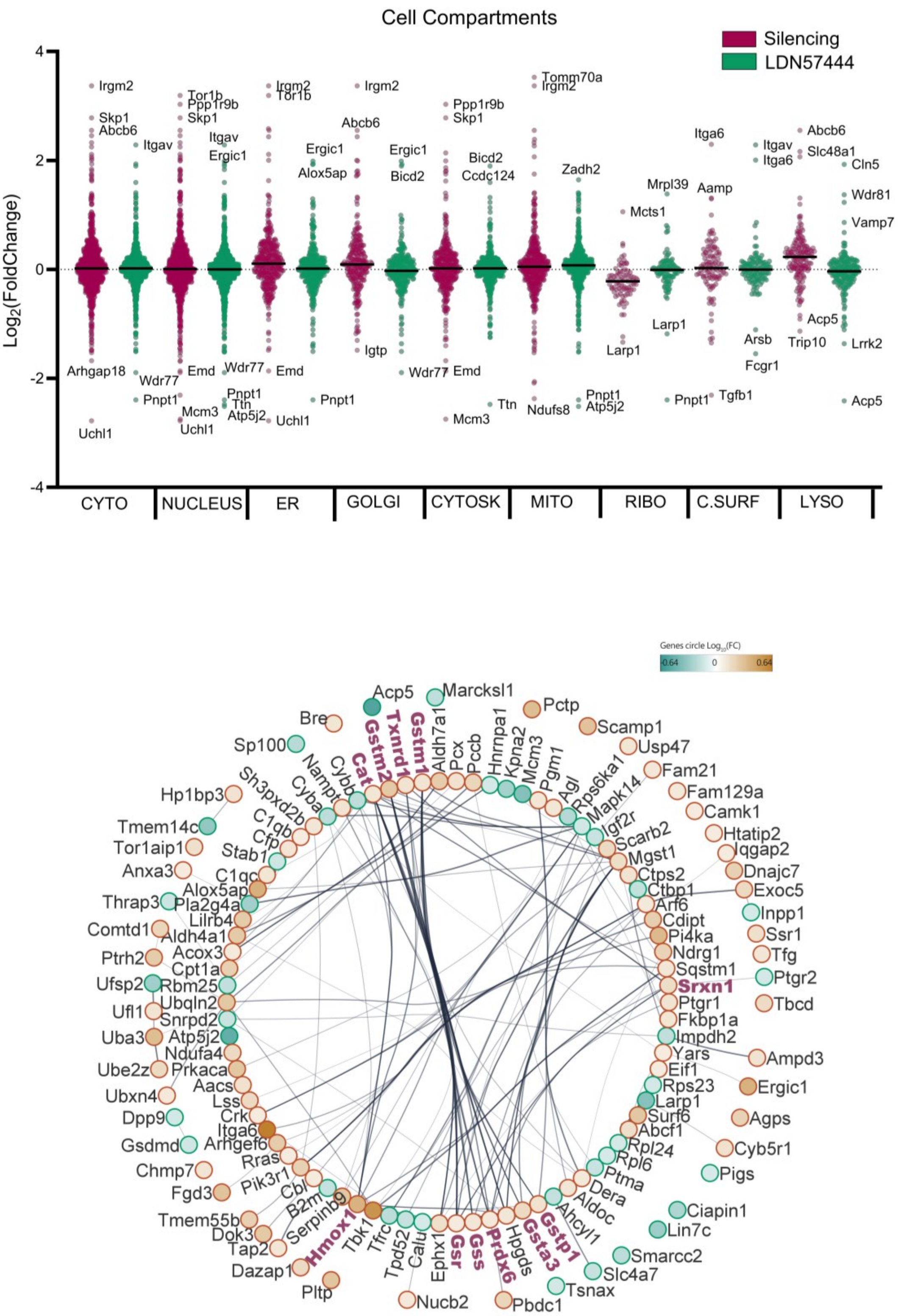

Supplementary Figure 4.

a) Proteins (gene names) quantified by label-free proteomic analysis in UCHL1-silenced (red) or pharmacologically inhibited (green) preOCs were grouped by the indicated GO Cell Compartments categories (average log_2 treated/control ratios); **b)** Proteins (gene names) consistently deregulated in both *Uchl1*-silenced and pharmacologically inhibited preOCs, highlighting gene products belonging to NRF2 response pathways (in purple) and overall expression ratios (average log_{10} treated/control ratios).
